# 100. Genomic epidemiology of carbapenemase-producing *Enterobacter* species in Toronto, Canada, 2007-2021

**DOI:** 10.1093/ofid/ofae631.037

**Published:** 2025-01-29

**Authors:** Conrad Izydorczyk, Robyn Lee, Laura Mataseje, George Golding, Tom Braukmann, Amanda C Carroll, Kevin Katz, Robert Kozak, Alainna J Jamal, Derek MacFadden, Tony Mazzulli, Roberto Melano, Samir Patel, Susan M Poutanen, David Richardson, Nicholas Waglechner, Allison McGeer

**Affiliations:** Sinai Health, Toronto, ON, Canada; McGill University, Montreal, Quebec, Canada; National Microbiology Laboratory, Winnipeg, Manitoba, Canada; Public Health Agency of Canada, Ottawa, Ontario, Canada; Ontario Agency for Health Protection and Promotion, Toronto, Ontario, Canada; The Ottawa Hospital Research Institute, Rockland, Ontario, Canada; North York General Hospital, Toronto, Ontario, Canada; Shared Hospital Laboratory, Toronto, Ontario, Canada; University of Toronto, Toronto, Ontario, Canada; The Ottawa Hospital Research Institute, Rockland, Ontario, Canada; Sinai Health System, Toronto, Ontario, Canada; Public Health Ontario Laboratory, Toronto, Ontario, Canada; Public Health Ontario, Toronto, Ontario, Canada; University Health Network and Sinai Health Network, Toronto, Ontario, Canada; William Osler Health System, Brampton, Ontario, Canada; Shared Hospital Laboratory, Toronto, Ontario, Canada; Mt. Sinai Hospital, Toronto, Ontario, Canada

## Abstract

**Background:**

Identification of transmission networks of carbapenemase-producing organisms (CPO) is critical to identifying their reservoirs and limiting their spread. This study aimed to use whole-genome sequencing to identify genomic relationships between CP *Enterobacter* (CP-Ent) in Ontario, Canada over a 15-year period.

**Figure:**
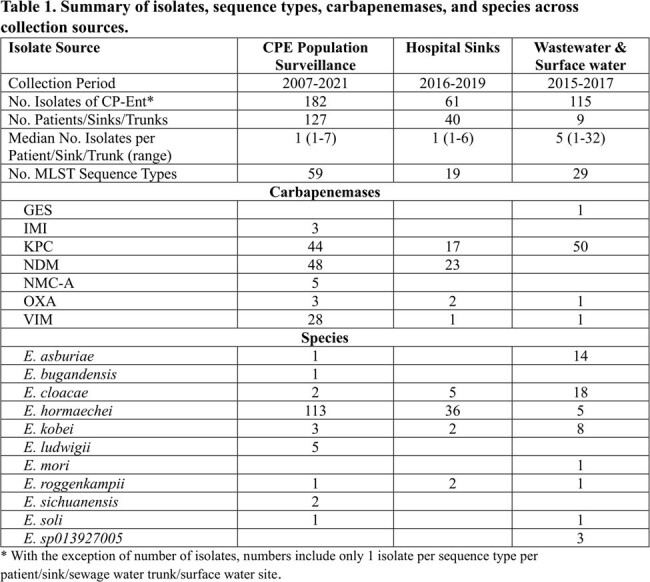

**Methods:**

All CP-Ent cases identified by prospective population-based surveillance in the Toronto/Peel Region from first isolate in 2007 to 2021, from hospital sinks (2016-2019), and wastewater treatment plants and surface water sites (2015-2017) were included. CPO isolates were identified by phenotypic screening (ertapenem MIC > 1mg/L or meropenem disc diffusion diameter ≤ 25mm), confirmed as *Enterobacter* by MALDI-TOF MS and as positive for carbapenemase genes by PCR, and sequenced by Illumina. Genomics analysis utilized a custom pipeline combining Snippy, IQ-Tree, and ClonalFrameML.

**Figure:**
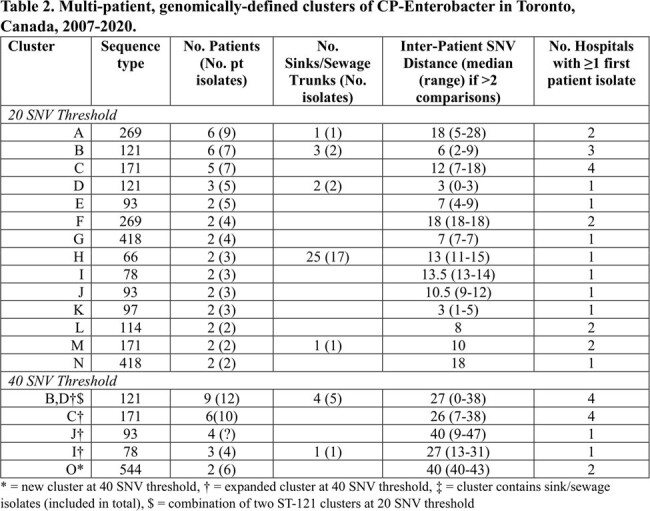

**Results:**

Overall, 182 isolates from 127 patients, 61 isolates from 40 sinks, and 155 isolates from 9 wastewater plants/surface water sites were available (Table 1). Thirty-one of 127 (24%) patients had > 1 sequenced isolate: in 29 patients all isolates were highly related (median SNV distance 3.5, range 0-13); one patient had two CP-Ent species ∼1.5 years apart, and one had 2 sequence types (STs) 86 days apart. Of 12 (30%) sinks with > 1 isolate, 5 had 2 STs recovered at different times (4 with different carbapenemases). Multiple species, STs, and carbapenemases were recovered from sewage trunks and surface water. Using a ≤ 20 SNV threshold between any one pair of isolates, 14 multi-patient putative clusters involving 39 patients were identified; 1 added cluster was identified and 4 expanded when the threshold was increased to 40 SNVs (Table 2). At the 40 SNV threshold, the median number of patients per cluster was 2 (range 2-9), and the median number of hospitals with at least one first identified patient in each cluster was 1.5 (range 1-4). In total, 35% (45/127) patients were part of a cluster.

**Conclusion:**

CP-Ent in Ontario are diverse, but a significant minority of affected patients are part of genomically-defined clusters. Genomic clusters may reflect undetected transmission of CP-Ent in healthcare, exposure to water, or other as yet unidentified sources.

**Disclosures:**

**Allison McGeer, MD**, AstraZeneca: Honoraria|GSK: Honoraria|Merck: Honoraria|Moderna: Honoraria|Novavax: Honoraria|Pfizer: Grant/Research Support|Pfizer: Honoraria|Roche: Honoraria|Seqirus: Grant/Research Support|Seqirus: Honoraria

